# A Cross-Sectional Study of Detection of Beta Globin (HBB) Haplotypes Among Beta Thalassemia Patients

**DOI:** 10.7759/cureus.13367

**Published:** 2021-02-16

**Authors:** Ali Alsamiri, Fatma Alzahrani, Najlaa Filimban, Ammar Khojah, Raed Felimban, Talal Qadah

**Affiliations:** 1 Medical Laboratory Technology, King Abdulaziz University - Faculty of Applied Medical Sciences, Jeddah, SAU; 2 Pediatrics, King Abdulaziz University Hospital, Jeddah, SAU

**Keywords:** beta thalassemia, beta globin gene, hbb haplotype

## Abstract

Introduction

Beta-thalassemia is among the most common monogenic disorders in the Arabian Peninsula. This study aimed to investigate the β-globin (HBB) haplotypes among β-thalassemia patients in Saudi cohort which have potential implications in understanding the clinical care of patients and population genetic factors associated with β-thalassemia.

Methods

We analyzed 60 β-thalassemia patients. Male/female distribution for β-thalassemia was 58.33%/41.66%. Results of hematological parameters and indices were obtained from the database. HBB haplotyping assay was performed for four specific loci of the HBB gene cluster using polymerase chain reaction-restriction fragment length polymorphism (PCR-RFLP) technique.

Results

HBB haplotyping assay identified three novel patterns namely haplotype 1, haplotype 2, and haplotype 3 and three common African haplotypes including Benin, Senegal, and Cameron. The frequency of haplotype 1 was the highest among the studied samples (62%, n = 37) with 56.76% (n = 21) observed in males compared to 43.24% (n = 16) in females. This was followed by Senegal, haplotype 2, Benin and haplotype 3 with similar percentage, and Cameron haplotype with 18%, 12%, 3% and 2%, respectively. The relationship between these haplotypes and various hematological parameters was calculated and our study found no significant relationship (p-value >0.05).

Conclusion

Our study indicated the importance of finding out types of β-globin haplotypes as novel types being discovered. Though no statistically significant association was identified among all the haplotypes in terms of hematological parameters, Cameroon or Benin haplotypes had the mildest form because they have the highest means among all parameters. Further studies need to be carried out on a larger population to detect the frequency of each specific mutation in each haplotype among β-thalassemia patients. This would help to re-address the question of the origin(s) of the β-thalassemia.

## Introduction

Thalassemia syndromes are a heterogeneous group of inherited anemias characterized by a defective biosynthesis of the alpha (α) or beta (β) globin subunits of the normal adult hemoglobin HbA (a2β2). This defect can cause the formation of abnormal hemoglobin molecules resulting in low hemoglobin level. This group of inherited disorders is relatively common worldwide and considered as the most common single-gene disorders [1]. It is commonly found in the Mediterranean area, Middle East, Equatorial Africa, and Southern Asia and the clinical severity varies greatly from asymptomatic to severe hemolytic anemia.

β-thalassemia is considered as one of the most common monogenic disorders in Saudi Arabia. This is in fact due to the high percentage of consanguineous marriages [2]. It constitutes one of the major public health issues encountered by the ministry of health due to its high impact on clinical management costs [3]. The prevalence of β-thalassemia in Saudi Arabia is estimated to be 1.36% where 0.07% represents the major type and 1.29% represents the trait (minor) type [4,5]. Various studies on the prevalence of β-thalassemia conducted over 20 years demonstrated that the Eastern province has the highest number of cases [6]. Molecular pattern of β-thalassemia showed a wide spectrum of mutations affecting the HBB and novel mutations are being discovered as the diagnostic tools are becoming more advanced [7]. It is noteworthy to mention that even though β-thalassemia is highly prevalent in the Eastern Province, a wide spectrum of clinical symptoms for this disorder is reported from the Western Province [8]. This variation could be contributed to the differences in the β-globin gene (HBB) haplotypes.

A haplotype can be defined as a set of genes of one chromosome that have been inherited together from a single parent [9]. HBB haplotypes settle on different combinations of polymorphic sites of HBB through the non-random link of the single point called restriction fragment length polymorphism (RFLP) [10]. Several mutations are found within and around HBB, resulting in different types of HBB haplotypes and thus causing population diversity [11]. The diversity in HBB haplotypes reflects the evolutionary and genetic relationship among this disorder in different ethnic backgrounds [12]. The importance of identification of HBB haplotype comes through the determination of DNA sequence variation across geographical populations. Those variations often caused by the transmission of genetic variations through populations from one generation to the next [13].

In Saudi Arabia, few studies have been performed to explore the molecular patterns of β-thalassemia [14-16]. In this study, we aimed to determine the HBB haplotypes among β-thalassemia patients in the Saudi cohort and correlate between these haplotypes and red cell parameters.

## Materials and methods

Sample collection and laboratory investigation

A total number of 60 blood samples from previously diagnosed β-thalassemia patients were obtained after consents were signed. The gender distribution included 25 (41.66%) females and 35 (58.33%) males. Hematological investigations were recorded through complete blood count (CBC) and Hb separation. The study was approved by the Ethics and Research Committee at Faculty of Applied Medical Sciences, King Abdulaziz University (Ref. No. FAMS-2019-003).

DNA extraction and preservation

DNA extraction was carried out using the Wizard® Genomic DNA Purification Kit (Promega, Madison, WI, USA) according to the manufacturer's instruction. The DNA concentration of the samples was assessed using Nanodrop 2000 spectrophotometer (Thermo Fisher Scientific, Waltham, MA, USA) and stored at -20°C for subsequent analyses.

β-globin (HBB) haplotyping assay

HBB haplotyping assay was performed for four specific loci of the HBB gene cluster using polymerase chain reaction-restriction fragment length polymorphism (PCR-RFLP) as previously described by Sutton et al. [17]. Briefly, the four loci were amplified by specific primer sets to generate PCR fragments of 655 bp, 771 bp, 766 bp and 388 bp for primer set numbers 1, 2, 3 and 4 respectively as shown in Table 1. These fragments contained sites for the restriction sites of XmnI (5' Gγ), HindIII (Gγ), HindIII (Aγ) and HinfI (5’/β) which were digested by the restriction enzymes. Identification of HBB haplotypes was determined through positive and/or negative reactions to digestion with restriction enzymes at these loci in order to generate specific patterns of a haplotype as demonstrated in previous works [18, 19].

**Table 1:** Sequence of primer, position and polymorphic site

Primers	Sequence	Position	Polymorphic site	Gene
1	F- AACTGTTGCTTTATAGGATTTT	33880	XmnI 34330	5' Gγ
	R- AGGAGCTTATTGATAACTCAGAC	34535		
2	F- TGCTGCTAATGCTTCATTACAA	35440	HindIII 35785	Gγ
	R- AAGTGTGGAGTGTGCACATGA	36211		
3	F- TGCTGCTAATGCTTCATTACAA	40371	HindIII 40731	Aγ
	R- TAAATGAGGAGCATGCACACAC	41137		
4	F- CTACGCTGACCTCATAAATG	60958	HinfI 61199	5/β
	R- CTAATCTGCAAGAGTGTCT	61341		

Statistical analysis

All statistical analysis in this study was carried out using either one-way ANOVA or Kruskal-Wallis test using SPSS, version 22 (IBM Corp., Armonk, NY, USA). A p-value < 0.05 was considered statistically significant. Regression analysis was used to study the association of HBB haplotypes with changes in the hematological parameters including red blood cell count (RBC), hemoglobin (Hb), hematocrit (Hct), mean cell volume (MCV), mean cell hemoglobin (MCH), mean cell hemoglobin concentration (MCHC), and red cell distribution width (RDW). Post hoc analysis was performed to find statistical significance among or in between the groups.

## Results

**Figure 1:**
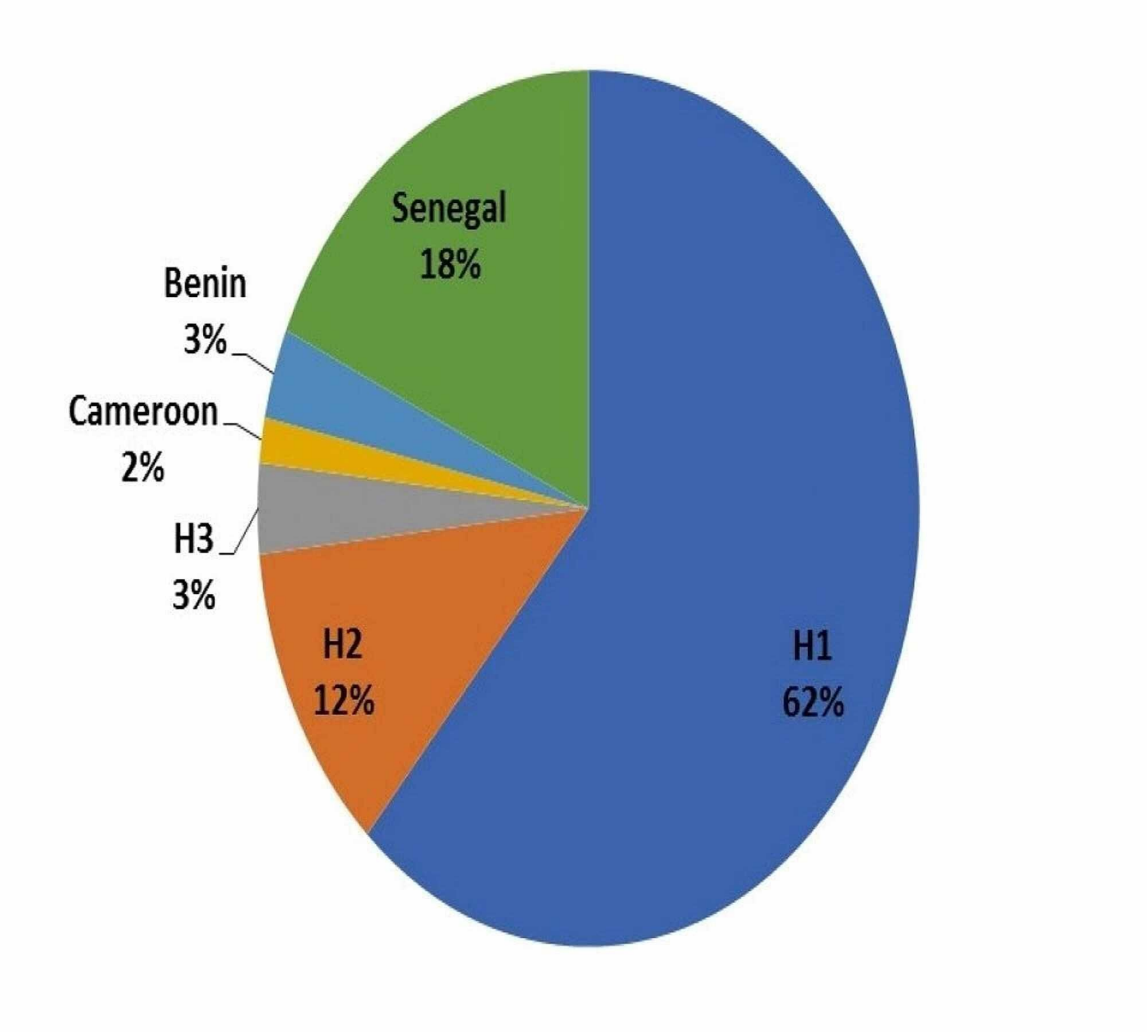
Frequency of HBB haplotypes. The pie chart shows the percentage of observed haplotypes HBB: β-globin

Recognition patterns of HBB haplotype generated from digestion of amplified regions with the restriction enzymes showed some common African haplotypes including Benin, Senegal, and Cameron while three other novel patterns namely haplotype 1, haplotype 2, and haplotype 3 were identified (Table 2). We found that the frequency of haplotype 1 was the highest with 62% (N = 37) with a mild predominance of males (56.76%, n = 21) compared to females (43.24%, n = 16). This was followed by Senegal haplotype (18%, N = 11), H2 (12%, N = 7), Benin (3%, N = 2), H3 (3%, N = 2) and Cameroon (2%, N = 1) as shown in Figure 1.

**Table 2:** Pattern of HBB haplotypes according to restriction enzyme HBB: β-globin

Haplotype	XmnI 5' Gγ	HindIII Gγ	HindIII Aγ	HinfI 5' β
Benin	-	-	-	-
Senegal	+	+	-	+
Cameroon	-	+	+	+
Haplotype 1	-	-	-	+
Haplotype 2	-	+	-	+
Haplotype 3	+	+	+	+

The association of red cell parameters among the various haplotypes of the cohort study was investigated and the results indicated that there is no statistically significant relationship between the haplotypes in terms of hematological parameters as all showed no significant scores (p-values above 0.05) (Table 3).

**Table 3:** Hematological parameters between haplotypes in thalassemic patients RBC: Red blood cell count; Hb: Hemoglobin; Hct: Hematocrit; MCV: Mean cell volume; MCH: Mean cell hemoglobin; MCHC: Mean cell hemoglobin concentration; RDW: Red cell distribution width.

Hematological parameters	Cameroon (N = 1, 1.66%)	Benin (N = 2, 3.33%)	H1 (N = 37, 61.66%)	H2 (N = 7, 11.66%)	H3 (N = 2, 3.33%)	Senegal (N = 11, 18.33%)	P-value KW
RBC (x 10^6^ /µL)	3.20	2.98 ± 0.21	2.99 ± 0.99	2.88 ± 1.30	2.26 ± 0.41	3.03 ± 0.63	0.644
Hb (g/dl)	10.4	9.60 ± 0.70	7.39 ± 2.51	5.85 ± 1.90	5.35 ± 1.48	7.17 ± 2.30	0.103
Hct (%)	30.2	26.55 ± 2.33	21.81 ± 7.53	19.07 ± 6.26	16.60 ± 4.10	21.93 ± 6.70	0.250
MCV (fL)	80.7	89.15 ± 1.48	72.34 ± 6.56	71.71 ± 16.74	73.20 ± 4.80	71.09 ± 10.32	0.183
MCH (pg)	27.60	32.10 ± 0.14	24.79 ± 4.71	24.24 ± 6.66	23.40 ± 2.26	23.19 ± 3.87	0.206
MCHC (g/dl)	34.30	36.00 ± 0.42	34.23 ± 5.22	33.52 ± 2.23	31.95 ± 1.06	32.56 ± 2.23	0.154
RDW	26.40	24.05 ± 3.75	25.32 ± 10.2	29.54 ± 5.95	34.10 ± 2.69	24.68 ± 8.42	0.706

Further, post hoc analysis was performed to see the statistical significance among and in between the haplotypes for each of the hematological parameters. As the Cameroon haplotype had just one sample, it was removed from the post hoc analysis. Though no statistically significant relationship between all the haplotypes in terms of hematological parameters were found.

Post hoc analysis showed significant difference between Benin and Haplotype 1 (p-value 0.012), Benin and Haplotype 2 (p-value 0.018), and Benin and Senegal (p-value 0.011) Haplotype for the MCV parameter. Similarly, significant difference was seen between BEN and Haplotype 1 (p-value 0.039), BEN and Haplotype 2 (p-value 0.044), and BEN and SEN (p-value 0.018) Haplotype for the MCH parameter (Table is attached in the appendices section).

**Figure 2:**
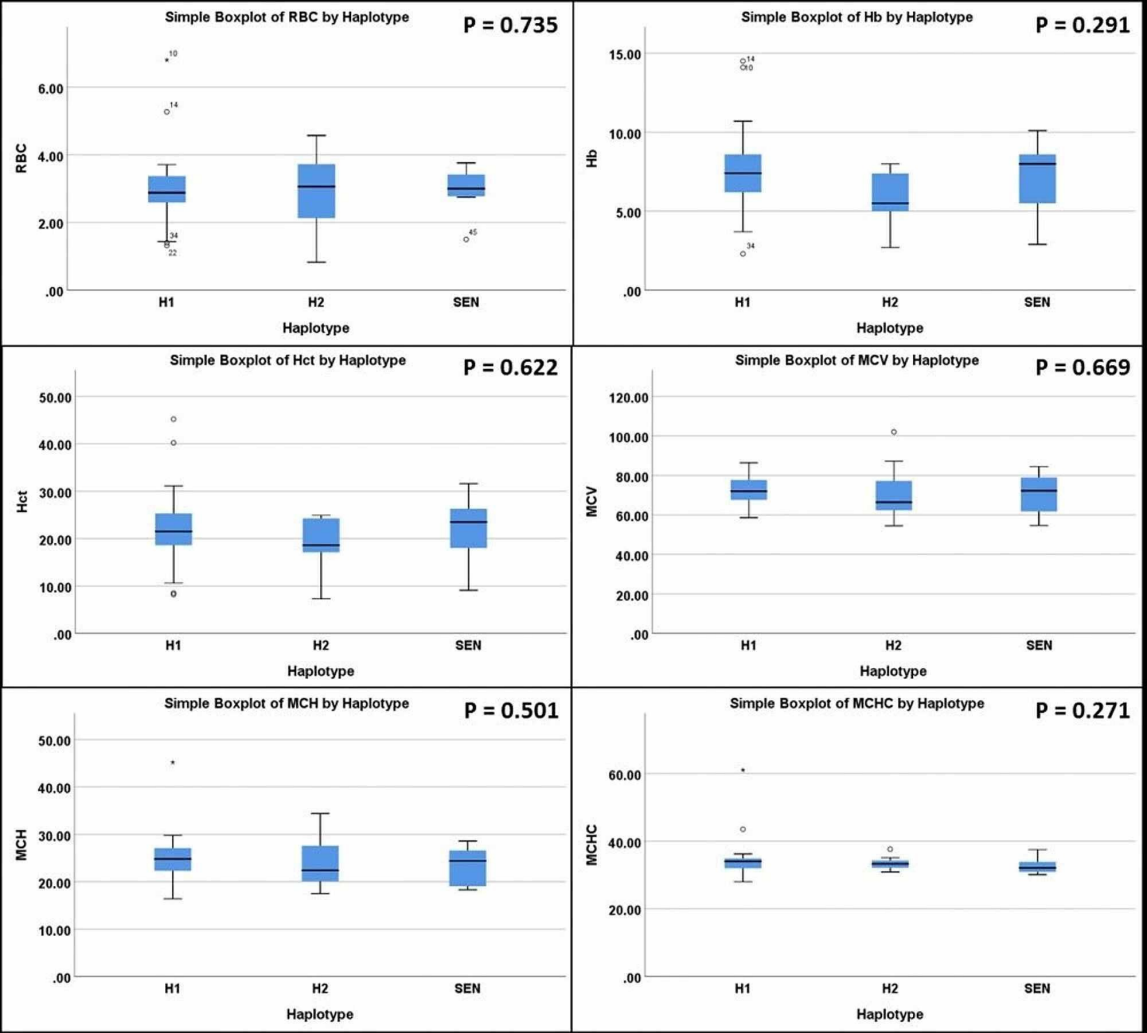
Comparison between the most frequent haplotypes (H1, H2, and Senegal) and the hematological parameters

To find out if the most frequent haplotypes from our cohort (i.e., H1, H2, and Senegal) have some effects on red cell parameters, we analyzed the relationship between them and found no significant association (Figure 2). The reason behind grouping H1, H2 and Senegal haplotypes was to find out if these haplotypes showed any differences in the red cell parameters, as together they formed the major number of samples (N = 55).

Further, a multinomial logistic regression analysis was used to analyze the association of HBB haplotypes with changes in the red cell parameters including RBC, Hb, Hct, MCV, MCH, and MCHC (Table 4). As can be seen from the significance (Sig.) column, there was no significant association between the changes in levels of the red cell parameters according to the haplotype. This indicates that red cell parameters are independent of HBB haplotype.

**Table 4:** Effect of each haplotype on RBC, HB, and HCT levels in thalassemia group using logistic regression model ^a^ The reference category is: SEN B – coefficient for the constant (intercept),  S.E. – Standard error around B, Sig. – Significance,  df –Degrees of freedom for the Wald chi-square test, Exp(B) – Exponentiation of the B coefficient, which is an odds ratio.

Haplotype^a^	Variable	B	Std. Error	Sig.	Exp(B)	95% Confidence Interval for Exp (B)	
						Lower Bound	Upper Bound
BEN	RBC	34.71803	486177.2	0.999943	1.2E+15	0	.b
CAM		-53.5923	65190.05	0.999344	5.31E-24	0	.b
H1		6.623199	5.313543	0.21259	752.3479	0.022569552	25079246.18
H2		393.0014	184556.5	0.998301	4.7E+170	0	.b
H3		-184.941	238438.9	0.999381	4.8E-81	0	.b
BEN	Hb	55.13301	175784	0.99975	8.79E+23	0	.b
CAM		3.35179	71006.04	0.999962	28.5538	0	.b
H1		3.287009	2.782163	0.237421	26.7627	0.114643263	6247.572354
H2		-115.81	69158.65	0.998664	5.06E-51	0	.b
H3		31.30258	254701.5	0.999902	3.93E+13	0	.b
BEN	Hct	-22.8203	85898.53	0.999788	1.23E-10	0	.b
CAM		6.54563	33057.49	0.999842	696.195	0	.b
H1		-2.01847	1.383707	0.144637	0.132859	0.008822198	2.000799385
H2		-20.9398	27749.34	0.999398	8.05E-10	0	.b
H3		14.9643	71759.27	0.999834	3154378	0	.b
BEN	MCV	-16.2453	12167.73	0.998935	8.81E-08	0	.b
CAM		-2.81946	15091.12	0.999851	0.059638	0	.b
H1		0.628306	0.695302	0.366184	1.874432	0.479763741	7.323388891
H2		38.89905	36003.64	0.999138	7.83E+16	0	.b
H3		57.65269	44585.15	0.998968	1.09E+25	0	.b
BEN	MCH	55.41141	63554.36	0.999304	1.16E+24	0	.b
CAM		3.435909	31252.32	0.999912	31.05962	0	.b
H1		-0.98305	1.863935	0.597912	0.374168	0.009693549	14.4428076
H2		-75.2429	95184.25	0.999369	2.1E-33	0	.b
H3		-169.735	126694.6	0.998931	1.93E-74	0	.b
BEN	MCHC	-46.6824	32312.69	0.998847	5.32E-21	0	.b
CAM		-2.6258	15729.97	0.999867	0.072382	0	.b
H1		0.430873	1.120979	0.700703	1.5386	0.17098069	13.84536686
H2		66.47738	65618.41	0.999192	7.43E+28	0	.b
H3		106.45	103533.7	0.99918	1.7E+46	0	.b

## Discussion

Our study was designed to determine the HBB haplotype among β-thalassemia patients and to analyze their effects on red cell parameters. Haplotype analysis has a statistical power because it reduces the association test dimensions. Rather than using genotyping individually, the haplotype analysis identifies common genetic features in a population [10]. Apart from this, several previous studies have shown that HBB haplotype analysis provides crucial information on genetic variations and haplotypes of different populations [20-22]. A study by Zhang et al., on genetic heterogeneity of* *HBB in various geographic populations of Yunnan in Southwestern China, identified seven different HBB haplotypes among 41 β-thalassemic chromosomes, where haplotype I and haplotype V were more than 65% (28 β-thalassemic chromosomes), while the remaining percentages were distributed to the other haplotypes [13]. Our study identified six haplotypes among 60 β-thalassemic patients from the Saudi cohort. The distribution of HBB haplotypes among β-thalassemic patients in our study showed that haplotype 1 was the highest with 62% (37 samples). This was in accordance with studies by various groups in the Mexican, Turkish and Western Iranian population which identified haplotype 1 to be the most prevalent haplotype among β-thalassemia chromosomes although restriction sites pattern is different [11, 21, 23]. Haplotype analysis of β-thalassemia patients was carried out in Western Iran by analyzing the pattern of seven restriction sites through the HBB gene cluster. It was found that haplotype I was the most prevalent haplotype with 35.7% among β-thalassemia chromosomes followed by haplotype III with 28.6% [11]. A previous haplotype analysis of three common β-thalassemia mutations performed in Syrian patients found out four HBB haplotypes among the studied patients where haplotype I was the most frequent one [23].

TheHBB haplotyping plays a role in the detection of severity level through observing the hematological parameters in hemoglobinopathies. The correlation between Arab-Indian haplotype and Benin haplotype indicated that the mean of RBC count was higher in Arab-Indian haplotype (3.9 million cells/mcL) than in Benin haplotype (3.0 million cells/mcL) indicating the milder form of this haplotype. Moreover, the mean Hb concentration was also higher in Arab-Indian haplotype (10.8 g/dl), than in Benin haplotype (8.4 g/dl). These differences in hematological parameters among the two haplotypes explained the diverse range in severity among HBB haplotypes [8]. In our study, the hematological parameters among the six haplotypes (Benin, Senegal, Cameroon, haplotype 1, haplotype 2, and haplotype 3) were investigated. The mean of RBC count was very close to about 3 million cells/mcL in all haplotypes except in haplotype 3 which was less than 2.5 million cells/mcL. The mean of Hb concentration in all haplotypes was above 7 g/dl, except haplotype 2 and haplotype 3. The mean was lowest in haplotype 3 with 5.35 g/dl. It was higher than 9 g/dl in Benin and Cameroon, which was consistent with Hb concentration for Benin haplotype in El-Hazmi findings [8]. The mean of Hct was almost similar for haplotype 2, haplotype 3 and Senegal at ~20%, whereas the lowest in haplotype 3 with 16.6% and greater than 25% in Benin and Cameron. Four haplotypes, haplotype 1, haplotype 2, haplotype 3, and Senegal showed similar MCV levels of nearly 72 fL, Benin and Cameroon had higher than 80 fL. Similarly, for MCH, haplotype 1, haplotype 2, haplotype 3, and Senegal showed similar levels at 24 pg, whereas Benin showed the highest with 32.1 pg and Cameroon at second highest at 27 pg. Five haplotypes showed a narrow range of MCHC level 32-34 g/dl, but Benin had the highest level at 36 g/dl. Although there was no significant alteration in red cell parameters among all haplotypes, we have observed that RBC parameters of haplotype 3 were the most affected ones since the anemia was more severe than others. Furthermore, HBB haplotypes are known to influence the severity of HBB diseases through hematological parameters [24]. A previous study about the effect of HBB cluster haplotype on hematological and clinical features of HBBmutation that cause sickle cell anemia was performed on 113 black American adults. The study found that Bantu haplotype usually is commonly associated with a severe form of the disease more than Benin and Cameroon haplotype where blood parameters in Benin and Cameroon haplotypes were characterized by mild clinical severity [25]. Though there is no Bantus haplotype in our study, we had a similar result for Benin and Cameroon haplotype where there was no significant difference between the patients. Additionally, all the haplotypes showed no significant difference among themselves in terms of the red cell parameters. However, when a pair wise comparison of significance was performed for each of the hematological parameters, we observed significant difference between Benin and Haplotype 1, Benin and Haplotype 2, and Benin and Senegal Haplotype for only MCV and MCH parameters, indicating that there is significant difference in severity of β-thalassemia for only the Benin, Haplotype 1, Haplotype 2 and Senegal haplotype as indicated from the hematological parameters MCV and MCH.

## Conclusions

Our study indicated the importance of finding out types of β-globin haplotypes as novel types being discovered. Though no statistically significant association was identified between all the haplotypes in terms of hematological parameters, but for MCV and MCH we identified in severity of β-thalassemia for only the Benin, Haplotype 1, Haplotype 2 and Senegal in the studied cohort. Our result is a preliminary analysis representing the distribution of β(S) haplotypes in the Western province of Saudi Arabia and future studies need to be carried out on a larger population to detect the frequency of each specific mutation in each haplotype among β-thalassemia patients. This would help to re-address the question of the origin(s) of the β-thalassemia.

## Appendices

**Table 5:** Post hoc analysis Post hoc analysis showed significant difference between Benin and haplotype I (p-value 0.012), Benin and haplotype 2 (p-value 0.018), and Benin and Senegal (p-value 0.011) haplotype for the MCV parameter. Similarly, significant difference was seen between BEN and haplotype 1 (p-value 0.039), BEN and haplotype 2 (p-value 0.044), and BEN and SEN (p-value 0.018) haplotype for the MCH parameter.

Multiple Comparisons							
LSD							
Dependent Variable	(I) Haplotype	(J) Haplotype	Mean Difference (I-J)	Std. Error	Sig.	95% Confidence Interval	
						Lower Bound	Upper Bound
RBC	Benin	Haplotype 1	-.01568	.69815	.982	-1.4154	1.3840
		Haplotype 2	.09857	.77106	.899	-1.4473	1.6445
		Haplotype 3	.72000	.96169	.457	-1.2081	2.6481
		Senegal	-.05273	.73925	.943	-1.5348	1.4294
	Haplotype 1	Benin	.01568	.69815	.982	-1.3840	1.4154
		Haplotype 2	.11425	.39638	.774	-.6804	.9089
		Haplotype 3	.73568	.69815	.297	-.6640	2.1354
		Senegal	-.03705	.33026	.911	-.6992	.6251
	Haplotype 2	Benin	-.09857	.77106	.899	-1.6445	1.4473
		Haplotype 1	-.11425	.39638	.774	-.9089	.6804
		Haplotype 3	.62143	.77106	.424	-.9245	2.1673
		Senegal	-.15130	.46497	.746	-1.0835	.7809
	Haplotype 3	Benin	-.72000	.96169	.457	-2.6481	1.2081
		Haplotype 1	-.73568	.69815	.297	-2.1354	.6640
		Haplotype 2	-.62143	.77106	.424	-2.1673	.9245
		Senegal	-.77273	.73925	.301	-2.2548	.7094
	Senegal	Benin	.05273	.73925	.943	-1.4294	1.5348
		Haplotype 1	.03705	.33026	.911	-.6251	.6992
		Haplotype 2	.15130	.46497	.746	-.7809	1.0835
		Haplotype 3	.77273	.73925	.301	-.7094	2.2548
Hb	Benin	Haplotype 1	2.20811	1.72417	.206	-1.2486	5.6649
		Haplotype 2	3.74571	1.90424	.054	-.0721	7.5635
		Haplotype 3	4.25000	2.37500	.079	-.5116	9.0116
		Senegal	2.42727	1.82568	.189	-1.2330	6.0875
	Haplotype 1	Benin	-2.20811	1.72417	.206	-5.6649	1.2486
		Haplotype 2	1.53761	.97890	.122	-.4250	3.5002
		Haplotype 3	2.04189	1.72417	.241	-1.4149	5.4986
		Senegal	.21916	.81562	.789	-1.4161	1.8544
	Haplotype 2	Benin	-3.74571	1.90424	.054	-7.5635	.0721
		Haplotype 1	-1.53761	.97890	.122	-3.5002	.4250
		Haplotype 3	.50429	1.90424	.792	-3.3135	4.3221
		Senegal	-1.31844	1.14830	.256	-3.6206	.9838
	Haplotype 3	Benin	-4.25000	2.37500	.079	-9.0116	.5116
		Haplotype 1	-2.04189	1.72417	.241	-5.4986	1.4149
		Haplotype 2	-.50429	1.90424	.792	-4.3221	3.3135
		Senegal	-1.82273	1.82568	.323	-5.4830	1.8375
	Senegal	Benin	-2.42727	1.82568	.189	-6.0875	1.2330
		Haplotype 1	-.21916	.81562	.789	-1.8544	1.4161
		Haplotype 2	1.31844	1.14830	.256	-.9838	3.6206
		Haplotype 3	1.82273	1.82568	.323	-1.8375	5.4830
Hct	Benin	Haplotype 1	4.73919	5.17925	.364	-5.6446	15.1230
		Haplotype 2	7.47857	5.72016	.197	-3.9897	18.9468
		Haplotype 3	9.95000	7.13429	.169	-4.3534	24.2534
		Senegal	4.62273	5.48417	.403	-6.3724	15.6178
	Haplotype 1	Benin	-4.73919	5.17925	.364	-15.1230	5.6446
		Haplotype 2	2.73938	2.94054	.356	-3.1560	8.6348
		Haplotype 3	5.21081	5.17925	.319	-5.1730	15.5946
		Senegal	-.11646	2.45005	.962	-5.0285	4.7956
	Haplotype 2	Benin	-7.47857	5.72016	.197	-18.9468	3.9897
		Haplotype 1	-2.73938	2.94054	.356	-8.6348	3.1560
		Haplotype 3	2.47143	5.72016	.667	-8.9968	13.9397
		Senegal	-2.85584	3.44938	.411	-9.7714	4.0598
	Haplotype 3	Benin	-9.95000	7.13429	.169	-24.2534	4.3534
		Haplotype 1	-5.21081	5.17925	.319	-15.5946	5.1730
		Haplotype 2	-2.47143	5.72016	.667	-13.9397	8.9968
		Senegal	-5.32727	5.48417	.336	-16.3224	5.6678
	Senegal	Benin	-4.62273	5.48417	.403	-15.6178	6.3724
		Haplotype 1	.11646	2.45005	.962	-4.7956	5.0285
		Haplotype 2	2.85584	3.44938	.411	-4.0598	9.7714
		Haplotype 3	5.32727	5.48417	.336	-5.6678	16.3224
MCV	Benin	Haplotype 1	16.80405^*^	6.49394	.012	3.7845	29.8236
		Haplotype 2	17.43571^*^	7.17215	.018	3.0564	31.8150
		Haplotype 3	15.95000	8.94524	.080	-1.9841	33.8841
		Senegal	18.05909^*^	6.87626	.011	4.2730	31.8452
	Haplotype 1	Benin	-16.80405^*^	6.49394	.012	-29.8236	-3.7845
		Haplotype 2	.63166	3.68696	.865	-6.7602	8.0236
		Haplotype 3	-.85405	6.49394	.896	-13.8736	12.1655
		Senegal	1.25504	3.07196	.684	-4.9039	7.4139
	Haplotype 2	Benin	-17.43571^*^	7.17215	.018	-31.8150	-3.0564
		Haplotype 1	-.63166	3.68696	.865	-8.0236	6.7602
		Haplotype 3	-1.48571	7.17215	.837	-15.8650	12.8936
		Senegal	.62338	4.32497	.886	-8.0477	9.2944
	Haplotype 3	Benin	-15.95000	8.94524	.080	-33.8841	1.9841
		Haplotype 1	.85405	6.49394	.896	-12.1655	13.8736
		Haplotype 2	1.48571	7.17215	.837	-12.8936	15.8650
		Senegal	2.10909	6.87626	.760	-11.6770	15.8952
	Senegal	Benin	-18.05909^*^	6.87626	.011	-31.8452	-4.2730
		Haplotype 1	-1.25504	3.07196	.684	-7.4139	4.9039
		Haplotype 2	-.62338	4.32497	.886	-9.2944	8.0477
		Haplotype 3	-2.10909	6.87626	.760	-15.8952	11.6770
MCH	Benin	Haplotype 1	7.30270^*^	3.44888	.039	.3881	14.2173
		Haplotype 2	7.85714^*^	3.80907	.044	.2204	15.4939
		Haplotype 3	8.70000	4.75074	.073	-.8247	18.2247
		Senegal	8.90909^*^	3.65192	.018	1.5874	16.2308
	Haplotype 1	Benin	-7.30270^*^	3.44888	.039	-14.2173	-.3881
		Haplotype 2	.55444	1.95811	.778	-3.3713	4.4802
		Haplotype 3	1.39730	3.44888	.687	-5.5173	8.3119
		Senegal	1.60639	1.63149	.329	-1.6646	4.8773
	Haplotype 2	Benin	-7.85714^*^	3.80907	.044	-15.4939	-.2204
		Haplotype 1	-.55444	1.95811	.778	-4.4802	3.3713
		Haplotype 3	.84286	3.80907	.826	-6.7939	8.4796
		Senegal	1.05195	2.29695	.649	-3.5532	5.6571
	Haplotype 3	Benin	-8.70000	4.75074	.073	-18.2247	.8247
		Haplotype 1	-1.39730	3.44888	.687	-8.3119	5.5173
		Haplotype 2	-.84286	3.80907	.826	-8.4796	6.7939
		Senegal	.20909	3.65192	.955	-7.1126	7.5308
	Senegal	Benin	-8.90909^*^	3.65192	.018	-16.2308	-1.5874
		Haplotype 1	-1.60639	1.63149	.329	-4.8773	1.6646
		Haplotype 2	-1.05195	2.29695	.649	-5.6571	3.5532
		Haplotype 3	-.20909	3.65192	.955	-7.5308	7.1126
MCHC	Benin	Haplotype 1	1.76216	3.22304	.587	-4.6997	8.2240
		Haplotype 2	2.47143	3.55965	.490	-4.6652	9.6081
		Haplotype 3	4.05000	4.43966	.366	-4.8510	12.9510
		Senegal	3.43636	3.41279	.318	-3.4059	10.2786
	Haplotype 1	Benin	-1.76216	3.22304	.587	-8.2240	4.6997
		Haplotype 2	.70927	1.82990	.700	-2.9595	4.3780
		Haplotype 3	2.28784	3.22304	.481	-4.1740	8.7497
		Senegal	1.67420	1.52466	.277	-1.3826	4.7310
	Haplotype 2	Benin	-2.47143	3.55965	.490	-9.6081	4.6652
		Haplotype 1	-.70927	1.82990	.700	-4.3780	2.9595
		Haplotype 3	1.57857	3.55965	.659	-5.5581	8.7152
		Senegal	.96494	2.14655	.655	-3.3386	5.2685
	Haplotype 3	Benin	-4.05000	4.43966	.366	-12.9510	4.8510
		Haplotype 1	-2.28784	3.22304	.481	-8.7497	4.1740
		Haplotype 2	-1.57857	3.55965	.659	-8.7152	5.5581
		Senegal	-.61364	3.41279	.858	-7.4559	6.2286
	Senegal	Benin	-3.43636	3.41279	.318	-10.2786	3.4059
		Haplotype 1	-1.67420	1.52466	.277	-4.7310	1.3826
		Haplotype 2	-.96494	2.14655	.655	-5.2685	3.3386
		Haplotype 3	.61364	3.41279	.858	-6.2286	7.4559
RDW	Benin	Haplotype 1	-1.27973	6.81036	.852	-14.9337	12.3742
		Haplotype 2	-5.49286	7.52162	.468	-20.5728	9.5871
		Haplotype 3	-10.05000	9.38110	.289	-28.8580	8.7580
		Senegal	-.63182	7.21131	.931	-15.0896	13.8260
	Haplotype 1	Benin	1.27973	6.81036	.852	-12.3742	14.9337
		Haplotype 2	-4.21313	3.86661	.281	-11.9652	3.5390
		Haplotype 3	-8.77027	6.81036	.203	-22.4242	4.8837
		Senegal	.64791	3.22164	.841	-5.8111	7.1069
	Haplotype 2	Benin	5.49286	7.52162	.468	-9.5871	20.5728
		Haplotype 1	4.21313	3.86661	.281	-3.5390	11.9652
		Haplotype 3	-4.55714	7.52162	.547	-19.6371	10.5228
		Senegal	4.86104	4.53571	.289	-4.2325	13.9546
	Haplotype 3	Benin	10.05000	9.38110	.289	-8.7580	28.8580
		Haplotype 1	8.77027	6.81036	.203	-4.8837	22.4242
		Haplotype 2	4.55714	7.52162	.547	-10.5228	19.6371
		Senegal	9.41818	7.21131	.197	-5.0396	23.8760
	Senegal	Benin	.63182	7.21131	.931	-13.8260	15.0896
		Haplotype 1	-.64791	3.22164	.841	-7.1069	5.8111
		Haplotype 2	-4.86104	4.53571	.289	-13.9546	4.2325
		Haplotype 3	-9.41818	7.21131	.197	-23.8760	5.0396
*. The mean difference is significant at the 0.05 level.							
